# Clinical experience of the importance of daily portal imaging for head and neck IMRT treatments

**DOI:** 10.1120/jacmp.v9i3.2756

**Published:** 2008-06-23

**Authors:** Laurence E Court, Luciant Wolfsberger, Aaron M Allen, Steven James, Roy B Tishler

**Affiliations:** ^1^ Department of Radiation Oncology Dana‐Farber / Brigham & Women's Cancer Center Boston MA USA

**Keywords:** image‐guided, localization, head and neck, IMRT

## Abstract

The purpose of this study was to evaluate patient setup in head and neck IMRT using daily portal imaging. At our institution, orthogonal digital portal images are taken daily to check patient positioning prior to head and neck IMRT treatment. Isocenter misalignments are corrected using a couch shift (3 mm action level). Therapists also compare the DRRs and portal images looking at points more distant from the isocenter, particularly in the supraclavicular region, and re‐position the patient's shoulders in the mask if considered necessary. The daily isocenter shifts (C2 region) and frequency of patient repositioning were investigated by review of record‐and‐verify records for 15 patients. The magnitude of the shoulder repositioning was evaluated for 10 of these patients by comparing portal images and plan DRRs for a point 8 cm inferior of isocenter (T2‐T4).

For all patients, pretreatment isocenter discrepancies 3 mm or smaller were recorded for a median of 92.5% of fractions (range: 71.4 – 100%). Patients were repositioned in the immobilization mask before treatment for a median of 14% of fractions (3–34%). Thirty percent of these were for shoulder shifts of 1 cm or larger. Twenty percent of patients needed shoulder shifts of 1 cm or more for more than 7/35 fractions, meaning that without setup based on daily imaging, parts of the CTV would have received less than 95% of the prescribed dose. In conclusion, with our current immobilization, isocenter positioning accuracy is excellent, while correct shoulder position is more variable, particularly for a small subset of patients. Frequent imaging of head and neck IMRT patients is essential to accurate delivery of therapy, with shoulder position an important factor.

PACS number: 87.53.Oq

## I. INTRODUCTION

When intensity modulated radiation therapy (IMRT) is used to treat tumors in the head and neck region, planning target volume (PTV) treatment margins are typically as small as 3–5 mm.^1^, ^4^ Target immobilization is usually achieved using head immobilization masks,^5^, ^12^ often combined with patient‐specific neck cushions. We found that in spite of the use of immobilization masks, the many degrees of freedom, shape change and large (long) targets in the head and neck region, make immobilization within these 3–5 mm criteria for the entire target volume a significant challenge. In particular, changes in the shape of the neck, and relative position of the shoulders (or supraclavicular region) can make achieving these goals difficult. Also, our experience showed that setup issues are particularly problematic for a relatively small sub‐set of patients. For these reasons, all head and neck IMRT patients at our clinic are treated using image‐guided radiation therapy techniques, where they are first positioned based on daily orthogonal portal images. We now have treated more than 300 patients using this technique. For this work we analyzed a subset of these patients to quantify our immobilization at isocenter (close to C2) and also in the supraclavicular region. This data will be useful as a benchmark of our current practice, allowing comparison with other immobilization techniques.

## II. METHODS AND MATERIALS

We retrospectively reviewed the records of 15 patients treated with IMRT for head and neck cancer at the Dana‐Farber Cancer Institute, with IRB approval. The treated sites covered all head and neck subsites including: nasopharynx, larynx, oral tongue, base of tongue, paraesophageal mass, paranasal sinus, thyroid, and unknown primary.

**Figure 1:**
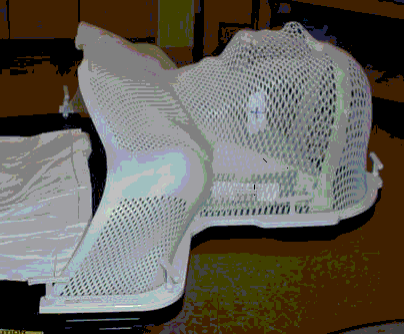
Head‐neck‐shoulder thermoplastic immobilization system (S‐Type, Medtec, Orange City, IA) and a custom constructed headrest (Acuform: Medtec).

Head and neck IMRT patients at our institution are immobilized using a head‐neck‐shoulder thermoplastic immobilization system (S‐Type, Medtec, Orange City, IA) and a custom constructed headrest (Acuform, Medtec), as shown in Fig. 1. Previous (anecdotal) experience at DFCI/BWH found that this gave better daily setup than with thermoplastic masks and standard head cups. For IMRT cases we use a generic isocenter, typically located around C2. All IMRT plans start with an imaging plan consisting of daily orthogonal portal imaging fields (6MV, 3MU per field). The imaging plan is included by the treatment planning system (Eclipse, Varian Medical Systems Inc., Palo Alto, CA) when optimizing the IMRT treatment. The final plan is considered as the sum of the imaging plan (which contributes approximately 2–3 Gy to the overall dose) and the IMRT plan (60–70 Gy, depending on the treatment site, and clinical situation). The CT images used for the plans and DRRs had 0.98×0.98 mm axial pixels, and 2.5 mm slice width.

In the treatment room, the patient is positioned in the immobilization mask. Before leaving the treatment room, the therapists carefully visually check the patient's positioning in the mask, and adjust if necessary. Orthogonal 6MV portal images are then taken using a digital portal imaging device (PortalVision, Varian Medical Systems Inc., Palo Alto, CA). Radiation therapist teams (2–3 therapists) visually compare the portal images to planned DRRs using Viewstation (IMPAC,

Mountain View, CA) to view the images. The therapists have full control over image display contrast, window level, image filter (smoothing filters etc.), and magnification. The DRR and portal image are displayed adjacent to each other, on the same screen. Based on comparison of AP and right lateral DRRs and portal images, isocenter misalignments are corrected by the therapists using a couch shift, with a 3 mm action level, prior to each fraction. This comparison is based on boney structures in the region of C2. Therapists also compare the DRRs and portal images looking at points more distant from the isocenter, particularly in the supraclavicular region. If there is poor agreement between the DRR and portal images for the supraclavicular region, then the therapists re‐enter the treatment room and physically reposition the patient in the mask. For the patient data analyzed here, we did not have a formal action level for repositioning the patient based on shoulder position. However, the therapists made this decision based on their knowledge that we planned using 5 mm margins. Because of the difficulty in judging shoulder movements, if a shoulder shift was needed the portal images were repeated and compared with the planned DRR to double check the actual amount the shoulders were moved. Similarly, we require re‐imaging for isocenter shifts 5 mm or larger, prior to daily treatment delivery. In summary, the flow is as follows:

1. Position patient in immobilization system.
2. Take AP and right lateral portal images.
3. Visually compare orthogonal portal images with orthogonal planned DRRs.
4. If the patient is not positioned correctly (i.e., if there are rotations that cannot be corrected with a couch shift), then reposition the patient in the mask and go back to [2].
5. If the patient is correctly positioned in the immobilization system, but there is a shift between portal images and DRRs larger than 3 mm, then move the patient couch to correct for the shift.
6. If the necessary isocenter shift was 5 mm or larger, go back to [2].

We evaluated the size and frequency of isocenter shifts in each orthogonal direction and lateral shoulder shifts. Isocenter shifts were collected for 15 patients (30–35 fractions per patient). The couch coordinates in record‐and‐verify records (IMPAC, Mountain View, CA) were reviewed for these patients, and the daily shift calculated as the change in couch coordinates recorded between the orthogonal images and the actual treatment fields. The frequency of shoulder shifts was also collected using therapists' notes in the patient record‐and‐verify chart for 10 of these patients. The size of shoulder shifts was not recorded in the chart, so was evaluated retrospectively by an experienced radiation therapist, who reviewed the original portal images and plan DRRs and noted the size and direction of the required shift for a point 8 cm inferior of isocenter. This is equivalent to T2–T4, and is close to the inferior edge of our head and neck IMRT fields (which include the supraclavicular region.)

Uncertainties in the isocenter shifts were estimated by asking a single therapist to independently review the isocenter position by comparing portal images and plan DRRs for 16 patient‐days (two patients). These shifts were compared with the shifts that were made clinically to estimate user‐dependent uncertainty in the isocenter shifts. The same images were used to estimate uncertainty in the shoulder shift data, where the user re‐reviewed the necessary shoulder shift two months after the original study. For this purpose, images were chosen which had been noted to require a shoulder shift in the patient chart.

## III. RESULTS

### A. Isocenter and shoulder shift results

For individual patients, isocenter discrepancies of 3 mm or smaller in any direction were recorded for 71.4 – 100% (median: 92.5%). Isocenter shifts larger than 5 mm were only recorded twice (2 patients, 1 posterior, 1 right, respectively; 0.38% of all fractions). Although individual patients often had a dominant direction of the shift, this was different for different patients. For example, for one patient, 83% of AP shifts were in the anterior direction, whereas for a different patient the situation was reversed, with 86% of AP shifts being made in the posterior direction. This was also true for R1 and SI shifts.

**Figure 2:**
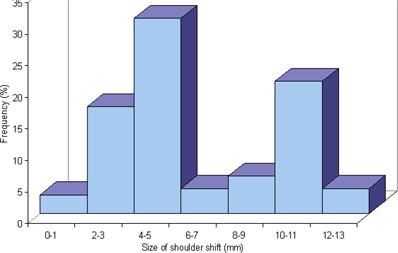
Size of shoulder shifts necessary (based on image review) for patients where shoulder shifts were originally made.

On the basis of pre‐treatment daily imaging, individual patients were repositioned in the immobilization mask before treatment for 3–34% of fractions (medial 14%, or 4.9/35 fractions). Fifty‐nine percent of these repositioning were for a shoulder shift of less than 5mm. Eleven percent of the shifts were from 6–9 mm. Thirty percent, however, were for shoulder shifts of 1 cm or larger. For each individual patient, all shoulder shifts of 1 cm or larger were in the same direction (left or right, depending on the patient). For the patients as a group, these were evenly divided between left and right. The worst case was a patient who had shoulder shifts 1 cm or larger for 11 out of 35 fractions (31%). For this patient, all large shoulder shifts were to the right. Fig. 2 shows the magnitude of the shoulder shifts. It can be seen that the distribution has two peaks. The first peak relates to small shifts (5 mm or less). The second shows large shifts centered at 1 cm.

### B. Estimate of uncertainties

The disagreement between the two groups for the isocenter shift was 0.3±2.1 mm (1s) for all directions. The 0.3 mm indicates a small systematic difference between the two groups. The standard deviation of 2.1 mm indicates the random differences in how the groups compared the DRRs and portal images. The maximum difference in the isocenter shift determined by the two groups was 3 mm in any one direction. That is, the two groups disagreed on the necessary shift by up to 3 mm. The standard deviation and maximum difference are fairly large, and for this data set led to disagreement between the groups of whether a shift was needed (using 3 mm criteria) for 35% of cases. That is, for 35% of cases there was disagreement whether the necessary shift was smaller or larger than 3mm.

The difference in the supraclavicular shifts found by the same user for the same patients when the images were reviewed two months apart was −1.8±2.9 mm. The 1.8 mm indicates a systematic difference in how the user interpreted the images. The maximum difference in the shift was 5 mm. This is worse than found for isocenter, but much smaller than the shoulder shifts found for the large shift peak (~1 cm).

## IV. DISCUSSION

PTV margins are a geometric concept, defined to ensure that, in the presence of setup and other uncertainties, the prescribed dose is actually delivered to the clinical target volume (CTV). For conformal head‐and‐neck treatments, many centers use an empirical PTV expansion of 5–10 mm based on historical norms and clinical experience. This expansion may have been appropriate for conventional treatment planning. However, with the increased use of IMRT a more exact understanding of the true uncertainty and corrections for it are necessary. There are several published formulations for calculating the necessary PTV.^13^, ^17^ For example, one formulation of van Herk^(14)^ showed that to ensure that the dose to the CTV is 95% for 90% of patients, the margin should be 2.5 times the systematic uncertainty plus 0.7 times the random uncertainty. For a systematic uncertainty of 1 mm, and random uncertainty of 2.5 mm, which is typical of those previously reported in the literature^7^, ^18^ for the head and neck region, this formula requires a margin of 4 mm. Another approach is to define margins based on radiobiological consideration, and this tends to require smaller margins.^(17)^ The many degrees of freedom and large (long) targets in the head and neck region mean that different portions of the target can move very differently to each other, and different anatomical regions can have different uncertainties. This makes immobilization within 3–5 mm criteria a significant challenge. In particular, even when localization at the isocenter is excellent, some patients exhibit large uncertainties in the supraclavicular region. These patients represent an important minority for whom margins based on the global population might not give sufficient coverage of the target.

One of the limitations of our study was the uncertainty related to visual interpretation of the images. The total (range) uncertainties were estimated less than 3 mm and 5 mm for isocenter and 8 cm inferior of isocenter, respectively. The effect of these uncertainties is to spread out the shifts reported here. Importantly, these uncertainties are smaller than the reported shifts for the supraclavicular region – which is the region with the larger, and therefore more important, shifts. We also made an assumption that the position of targets or avoidance structures relative to bony landmarks is consistent fraction to fraction. This assumption, which has been made by other researchers,^7^, ^8^, ^18^ seems reasonable given the close proximity of all the structures in the head and neck region.

Another limitation is the use of 2D/2D matching to determine the accuracy of patient setup. This is the traditional technique, and is also the only technique available in most clinics. However, there is an important flaw in this technique because it is not always possible to distinguish mismatches due to translational shifts from those due to rotations. For example, a rotation of the head can appear as a shift when viewing the AP image. Similarly, a yaw (rotation about the anterior‐posterior direction) will appear as a translation for the supraclavicular region. The appropriate corrective action is, in this case, somewhat unclear, as it is unlikely that the entire head and neck region will be subject to exactly the same yaw. That is, the head is better immobilized than the neck and may move differently. The approach taken in our clinic is to correct for these types of setup errors by repositioning the patient in the immobilization system so that all setup errors can be corrected with shifts alone. Although it may be argued that a couch rotation is sufficient to correct for these errors in the positioning of the supraclavicular region, this requires further study (including dosimetric analysis) with multiple CT images of the patient, and is beyond the scope of this work.

Our results for isocenter shifts are consistent with results recently presented by Zhang et al., ^(18)^ who, based on analysis of daily CT images, reported a 90% confidence interval of 3.2 – 3.8 mm for C2. They reported no significant difference when using the S‐board or conventional face mask. Our results for T2–T4 (8 cm inferior of isocenter), for which we found shifts smaller than 5 mm for 94% of all fractions, were also similar to those of Zhang et al., who reported a 90% confidence interval of 5.2 mm in the RL direction for C6. Similarly, for similar thermoplastic masks, Gibleau^(7)^ found uncertainties (1 standard deviation) of 1.8 mm in the head and neck region, and 2.4 mm in the shoulders.

**Figure 3:**
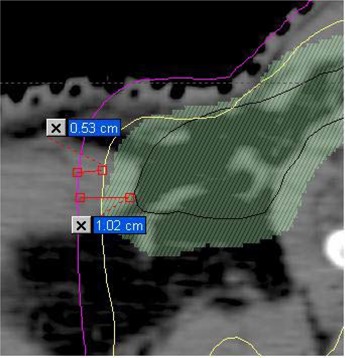
A detail of an IMRT plan at the shoulder level showing the physician‐drawn CTV (black contour), PTV after 5 mm expansion (green shaded area), 100% isodose line (yellow) and 75% isodose line (purple). Also shown are measurements indicating that the CTV is 1 cm away from the 75% isodose line, and that there is 0.5 cm between the 75% and 100% isodoses.

A particularly important result of this work is that we have identified the presence of dosimetrically significant outliers for the shoulder position that have not been reported in other studies. Although 94% of all fractions across all patients showed shoulder shifts less than 5 mm, a small group of patients required a significant number of large (1 cm) shoulder shifts. A review by the authors of typical head‐and‐neck IMRT plans (5 mm margins) at our institution showed that a 1 cm lateral shift in the shoulder region can reduce the dose to the CTV by as much as 25% or more. An example is shown in Fig. 3, which shows a detail from an IMRT plan at the level of the shoulders. It can be seen that a 5 mm shift would move the volume covered by the 100% isodose to the 75% isodose line. A 1 cm shift would move the physician‐drawn CTV to the 75% isodose line. This means that, to keep any cumulative dose reduction to any point of the CTV to less than 5%, no more than 7 out of 35 fractions (20%) can have 1 cm shifts (in the same direction). If we had not used daily imaging to check setup, this would not have been met for 20% (2/10) of our patients. Daily imaging, therefore, is important to prevent underdosing of the targets. The setup uncertainty does not present an issue for cord dose, partly because we use a 7 mm margin around the cord, and (more importantly) because most of the targets in the supraclavicular region are anterior to, and not adjacent to, the cord.

We have noted that it is not possible to predict which patients will experience large shoulder shifts nor do we have a strategy for identifying those patients. In addition, no significant shoulder shifts in the first week of treatment does not necessarily correspond to few/no shoulder shifts later in that patients treatment. One patient had no shoulder shifts larger than 5 mm until their 32^nd^ fraction, but had large shifts for every fraction thereafter (total treatment: 35 fractions). Other patients had large shifts from the very start of treatment. This would suggest that daily imaging is necessary throughout the entire course of therapy for head and neck IMRT patients in order to have confidence in our dosimetry for each and every patient.

Since one significant source of uncertainty is movement of the supraclavicular region, an approach taken by some investigators is to treat the supraclaviclular region using a conventional anterior field. However, recent work of Thorstad et al.,^(19)^ indicated that a cold match line between conventional and IMRT fields can be a significant cause of recurrences. Another option would be to use larger margins for targets in the supraclavicular region. However, for the majority of patients, this would mean an unnecessary increase in dose to normal tissue. We are also looking at alternative immobilization techniques to improve setup uncertainty in the shoulder region. Alternatively, because we use daily imaging it might be argued that we could reduce our PTV margin from 5 mm to 3 mm or smaller. However, because of the size of the user uncertainties from visual evaluation of the data, and other uncertainties indicated above, together with the shape change that we notice for these large (long) targets (e.g., neck yaw and tilt) mean that we are currently maintaining use of 5 mm margins in the clinic. Also, we have not investigated intra‐fraction motion, although this is usually less than 1 mm, depending on details of the immobilization device used.^(5)^

An important result of this work is that we have demonstrated that digital portal imaging, which is available in most radiation therapy centers, can be used clinically on a daily basis to correct daily patient setup. Our use of daily imaging is straightforward and only adds approximately three minutes to each fraction. Although global population data might provide an ability to create a class solution for PTV expansion, we have shown that there is an important minority who would not receive the planned dose distribution if we did not use daily imaging to check positioning immediately before treatment of each fraction.

## V. CONCLUSIONS

When thermoplastic head‐neck‐shoulder immobilization and customized headrests are used, isocenter positioning accuracy can be excellent, but shoulder positioning is more variable. For some patients, this variability can be dosimetrically important. Frequent imaging of head and neck IMRT patients is important to accurate delivery of therapy. It is essential for the imaging field size to show the entire treatment area, as shoulder position is an important factor and the most variable aspect of positioning. Weekly imaging, while giving some feedback on positioning, does not identify these variations in positioning.
